# CNS Vasculitis in Anti-Synthetase Syndrome

**DOI:** 10.31138/mjr.30.4.220

**Published:** 2019-12-31

**Authors:** Muhammad Shipa, Maria Di Cicco, Euthalia Roussou

**Affiliations:** Department of Rheumatology, Barking Havering and Redbridge University Hospitals NHS Trust, Romford, United Kingdom

**Keywords:** anti-synthetase syndrome, antibodies, CNS, vasculitis

## Abstract

A 52-year-old woman known to have anti-synthetase syndrome (ASS) with positive anti-alanyl-tRNA synthetase antibody (anti-PL 12) for 4 years presented with headache and progressive deterioration of cognitive functions manifested predominantly by episodes of confusion and dyslexia. Clinical, laboratory and radiological evaluation as well as response to treatment was indicative of vasculitis of the central nervous system (CNS). CNS vasculitis is one of the rare manifestations of inflammatory myositis and no case has been reported to suggest CNS vasculitis in ASS.

## INTRODUCTION

Anti-synthetase syndrome (ASS) is a rare, autoimmune disease of unknown aetiology. This encompasses inflammatory myositis, interstitial lung disease, articular and oesophageal involvement, Raynaud’s phenomenon, mechanic’s hands and presence of anti-synthetase auto-antibodies.^1^ Anti-JO-1 antibody is the most common autoantibody identified,^2^ but other anti-synthetase antibodies, anti-alanyl-tRNA synthetase (anti-PL 12) and anti-threonyl-tRNA (anti-PL 7) antibodies, have also been found to be present.^3^ ASS associated with anti-PL12 antibodies is a rare condition; hence, the specific characteristics of anti-PL12-associated ASS are not well described. A case series consisting of 17 patients with anti-PL12 associated ASS demonstrated 100% lung involvement, whereas only 41% of them had myositis, dermatomyositis being the most common histological variety (76%).^4^ Oesophageal involvement (23.5%) and pulmonary hypertension (23.3 %) have also been noted.^4^ Another case series from a total of 31 patients supports similar findings describing 90% of lung involvement and 46% of muscle involvement.^5^ Central nervous system (CNS) involvement has not been described in ASS before. We report the case of a 52 years old lady with anti-PL 12 associated ASS who developed CNS vasculitis.

## CASE PRESENTATION

**Figure 1.**
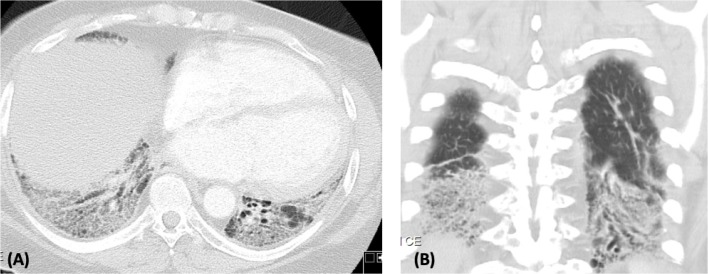
Computer Tomography (CT) axial view (a) and coronal view (b) show honeycombing and lower lobe ground glass opacities suggesting interstitial lung disease.

A 52-year-old woman of African descent who initially presented with polyarthralgia and knee pain following an episode of chest infection (with fever) which was treated as pneumonia was subsequently found to have interstitial lung disease. X-rays of hands and feet were unremarkable. While she was admitted for her pneumonia, urine test showed red cell casts, hyaline casts, granular casts, as well as haematuria, but no proteinuria. Subsequent urine tests following the admission were clear from casts. Laboratory investigation showed high globulin levels with raised IgG and IgA. Rheumatoid factor (RF), antinuclear antibodies (ANA), extractable nuclear antibodies (ENA), anti-double stranded DNA antibodies, anti-neutrophil cytoplasmic antibody (ANCA) MRO and PR3, ACE levels and myeloma screen were all negative. The immunology laboratory sent an extra report stating that she had positive myositis antibodies anti PL-12 with negative anti-Jo1, PL-7, SRP antibody, OJ antibody, EJ antibody, anti-Ku, anti-Mi-2, anti MDA-5 and PM-Scl antibody. The patient therefore had been diagnosed as anti-PL12-associated ASS. She also had the following relevant clinical and pathophysiological findings: a) Lung fibrosis: High resolution computed tomography (HRCT) of the lungs showed honeycombing and lower lobe ground glass opacities (*Figure 1a, Figure 1b*). Diffusion capacity for carbon-monoxide (DLCO) was 2.8 mmol/min/kPa which was 38% of the predicted value. The vital capacity was reduced moderately, however there was no airflow obstruction on spirometry. Moreover, she desaturated at the 6-minute walk test (6MW). b) Inflammatory myositis: Biopsy results showed dermatomyositis pattern on immunohistochemistry with perivascular, peri-fascicular inflammations and positive CD4 T-cell with muscle fibre phagocytosis and necrosis along with expression of major histocompatibility complex (MHC)-class I antigen. Neurophysiology studies were indicative of myopathy. (c) Pulmonary hypertension: cardiac catheterization showed mean pulmonary arterial pressure (mPAP) of 56 mmHg with pulmonary capillary wedge pressure (PCWP) of 5 mm Hg and pulmonary vascular resistance (PVR) of 16.7 wood units. (d) Dysphagia, which was steroid responsive. Endoscopy and colonoscopy did not reveal any other abnormalities from the gastroenterology system.

Other conditions not related to ASS but presented here for completeness included herpes labialis, irritable bowel syndrome, bowel obstruction due to adhesions from a hysterectomy for fibroids for which she had an operation, and no evidence of vasculitis had been identified.

She was stable for a number of years on azathioprine (125 mg daily), prednisolone (5 mg daily) along with macitentan (10 mg daily) and tadalafil.

When presented to the rheumatology clinic, she described recent onset of generalized, intermittent and moderate to severe intensity headache without any aura, nausea or vomiting. Additionally, she showed deterioration of cognitive function. The neurologist who assessed her described her being “emotional” in recent months and “having difficulty in word finding” with no clinical evidence of dementia. In the clinical notes, there is a description of relapsing episodes of confusion (during one of which she missed a return flight home from a trip abroad) and psychosis, and a referral to a consultant neuropsychologist took place from the neurology department. Full neuropsychiatric examination suggested a Montreal Cognitive Assessment (MOCA) score of 22 out of 30 with depressed visuospatial and abstraction functions along with dyslexia. Full neurological examination including fundoscopy showed no other abnormalities.

Laboratory investigations showed raised erythrocyte sedimentation rate (ESR) of 86 mm/h (normal value 0–22 mm/h), C- reactive protein (CRP) of 44 mg/L (normal values <5 mg/L) and lactate dehydrogenase of 519 U/L (normal values 150–280 U/L). Full blood count, liver, renal and bone profiles, complements levels, protein electrophoresis and amyloid A levels were all within normal limits. ANA, anti-ds DNA, ENA, RF, ANCA and anti-phospholipid antibodies were also negative. Additionally, the patient has been checked for Human Immunodeficiency Virus, Hepatitis B and C, Tuberculosis and Syphilis screening, which were all negative.

**Figure 2.**
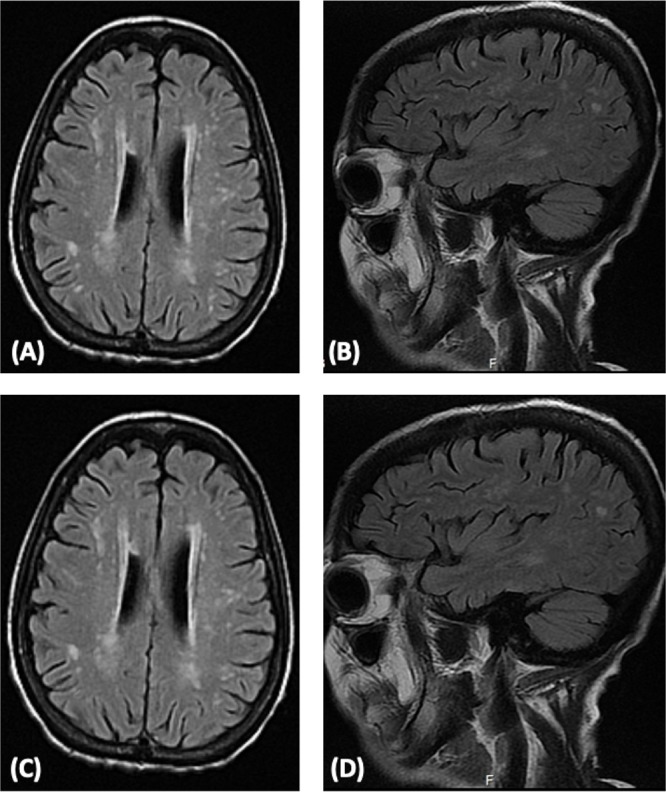
MRI Brain axial view (a) and sagittal view (b) T2 FLAIR suggest hyper-intense lesions in cortical, subcortical, periventricular and corpus callosum. MRI Brain axial view (c) and sagittal view (d) T2 FLAIR suggest no progression of the above mentioned hyper-intense lesions.

**Figure 3.**
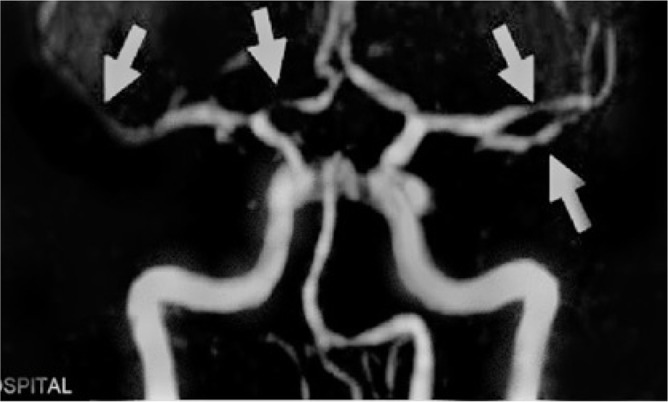
MRA of multiple areas of stenosis (irregular tapering and narrowing) suggesting vasculitis.

An MRI of brain demonstrated widespread nonspecific T2, fluid attenuation inversion recovery (FLAIR) high-signal intensity lesions in cortical, subcortical, deep periventricular white matter, optic radiation and corpus callosum of both hemispheres (*Figure 2a* and *Figure 2b*). Magnetic resonance angiogram (MRA) of brain with contrast showed multiple areas of irregular tapering and narrowing of middle and anterior cerebral artery without any beading appearance (*Figure 3*). The radiological (MRI and MRA) findings were discussed at the multidisciplinary neuroradiology meeting and the consensus opinion was that the signal abnormalities in her brain were due to ischaemic changes in small and medium sized blood vessels rather than from multifocal demyelination.

Cerebrospinal fluid (CSF) analysis revealed mild pleocytosis (50/mm^3^) with predominant lymphocytes (95%) along with elevated CSF protein (65 mg/dl) and normal glucose. Lymphocyte analysis revealed predominance of T cells (80%) with fewer mature B cells (13%), and no clonal B cells. Immunoglobulin IgG was slightly raised (13 mg/dL; normal values 0.0–8.1 mg/dL) with no oligoclonal band. Extensive investigation for infections was negative. Based on the above findings CNS vasculitis was diagnosed. Upon diagnosis, azathioprine was stopped and the patient was treated with intravenous pulses of methylprednisolone 1 g for three consecutive days, followed by oral prednisolone (started from 60 mg/day and tapered down to 10 mg/day over 6 months) along with oral cyclophosphamide (1.5 mg/Kg/day) for six months. The above regime was given as an induction therapy. After the induction therapy, while she remained clinically and biochemically stable (improvement of headache and dyslexia, and normalisation of inflammatory markers -ESR 12 mm/hr and CRP <5 mg/dl), she was switched to a maintenance treatment using Mofetil mycophenolate (MMF–2 g/day) prednisolone (10 mg/day), which maintained the remission over the subsequent 24 weeks. Repeat MRI of brain at 24 weeks, confirmed stable appearances without any further progression (*Figure 2c* and *Figure 2d*).

MOCA calculated at 6 months was 24 out of 30 (it was 22/30 before) with improvement of depressed visuo-spatial function. There was no further deterioration of abstraction functions or dyslexia.

Upon scrutinising the notes, it became evident that the patient was able to identify CNS symptoms in the form of confusion which she herself had attributed to the reduced dose of prednisolone. (Lower than 10 mg daily).

## DISCUSSION

To the authors’ knowledge, this is the first described case of CNS vasculitis in anti-PL-12-associated myositis. The diagnosis of CNS vasculitis can be challenging, particularly in a non-typical clinical setting. In our case, ASS had been established, and the patient was well for 4 years prior to the onset of cognitive dysfunction and dyslexia and while on treatment with azathioprine she developed CNS manifestations responding to treatment with cyclophosphamide and prednisolone which is the standard treatment for CNS manifestations in systemic lupus erythematosus and other vasculitides. Other causes of CNS involvement, such as infections were excluded. MRI and MRA of brain, although non-specific, were well supportive of the diagnosis of vasculitis. Other autoimmune conditions and vasculitides typically associated with CNS involvement were ruled out on the basis of the negative autoantibody panel and negative relevant clinical and laboratory features. The presence of cellular casts and haematuria was found on intermittent urine samples during the admission with pneumonia. However, given the on/off positivity, the lack of proteinuria and the normal renal function tests, no specific concern for renal vasculitis raised in the patient. Although the gold standard for the diagnosis of CNS vasculitis is leptomeningeal brain biopsy, we did not proceed with biopsy to get histopathological confirmation due to the difficulties in obtaining brain biopsies. However, all the investigations as well the clinical presentation and the response to treatment pointed out to the accuracy of the diagnosis of CNS involvement.

We appreciate that the pathogenetic origin of the CNS involvement is difficult to establish in this patient. CNS vasculitis has been exceptionally reported in few cases of dermatomyositis and Juvenile onset dermatomyositis,^6–10^ but not in ASS. It is not known whether CNS involvement could be a distinctive feature of anti-PL-12 antibody-associated ASS – driven by a specific tropism of these antibodies for the cerebral tissue - or whether CNS involvement may represent another subtype of ASS. Any possible pathogenetic role of myositis antibodies in the development of neurological manifestations is unknown. Detection of anti-PL-12 in the cerebral fluid could have been helpful. However, this test was not readily available in our laboratory.

Based on these findings, in the presence of new onset neurological symptoms not explained otherwise within the context of ASS, a potential involvement of the CNS system by the underlying autoimmune condition should be suspected and investigated, as a prompt diagnosis and the start of immunosuppressant therapy are essential for a favourable outcome in these patients.
